# Tracing internal quality and aroma of a red-fleshed kiwifruit during ripening by means of GC-MS and E-nose

**DOI:** 10.1039/c9ra03506k

**Published:** 2019-07-08

**Authors:** Dongdong Du, Min Xu, Jun Wang, Shuang Gu, Luyi Zhu, Xuezhen Hong

**Affiliations:** College of Biosystems Engineering and Food Science, Zhejiang University Hangzhou 310058 PR China jwang@zju.edu.cn +86-571-88982191 +86-571-88982178; Key Laboratory of On Site Processing Equipment for Agricultural Products, Ministry of Agriculture and Rural Affairs Hangzhou 310058 PR China; College of Quality & Safety Engineering, China Jiliang University Hangzhou 310018 PR China

## Abstract

‘Hongyang’ kiwifruit is a new breed of red-fleshed cultivar that has become broadly popular with consumers in recent years. In this study, the internal quality and aroma of this kiwifruit during ripening were investigated by means of gas chromatography-mass spectrometry (GC-MS) and electronic nose (E-nose). Results showed that the green note aldehydes declined, the main fruity esters increased, and the terpenes had no obvious changes during ripening. Correlations between quality indices, volatile compounds, and E-nose data were analyzed by ANOVA partial least squares regression (APLSR), and the results showed that firmness and titratable acidity (TA) had highly positive correlations with (*E*)-2-hexenal and hexanal, while soluble solids content (SSC) and SSC/TA ratio had positive correlations with ester compounds. The E-nose sensors of S7, S10, S8, S6, S9, and S2 were positively correlated with ester compounds, S1, S3, and S5 were mainly correlated with hexanal, and S4 was correlated with terpene compounds. Partial least squares regression (PLSR) and support vector machine (SVM) were employed to predict the quality indices by E-nose data, and SVM presented a better performance in predicting firmness, SSC, TA, and SSC/TA ratio (*R*^2^ > 0.98 in the training set and *R*^2^ > 0.94 in the testing set). This study demonstrated that the E-nose technique could be used as an alternative to trace the flavor quality of kiwifruit during ripening.

## Abstract 2

‘Hongyang’ kiwifruit is a new breed of red-fleshed cultivar that has become broadly popular with consumers in recent years.

## Graphical abstract

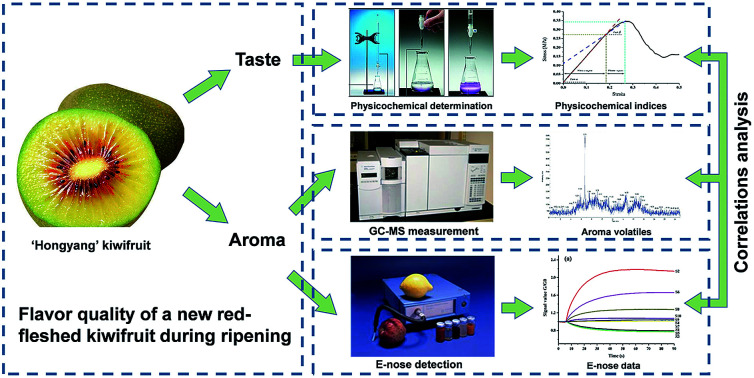

## 1 Introduction

Kiwifruit is one of the most valuable fruits, which is native to China and popular around the world. There are more than 70 cultivars of kiwifruit, of which only a few are of commercial importance, mainly *Actinidia deliciosa* and *Actinidia chinensis*. The green-fleshed kiwifruit ‘Hayward’ is the most renowned, occupying about 90% of the kiwifruit in the global market.^1^ ‘Hort16A’ is a yellow-fleshed kiwifruit that is the first *A. chinensis* to be commercialized outside China. After 20 years of selection and breeding, a new red-fleshed kiwifruit (*Actinidia chinensis* Planch. var. rufopulpa [C. F. Liang and R. H. Huang] C. F. Liang and A. R. Ferguson) cultivar called ‘Hongyang’ was released in China.^2^ Then ‘Hongyang’ kiwifruit become widely popular with consumers, not only because of the appealing flesh with red pigments in the inner pericarp, but also because of the distinctive flavor.^3^

Fruit and vegetable flavor mainly depends upon taste and aroma.^4^ Previous studies on the flavor quality of kiwifruit mainly focused on the two commercial cultivars ‘Hayward’ and ‘Hort16A’.^5^ Firmness, sweetness, and acidity are considered as the most important taste-related internal quality attributes to consumers.^6^ It is reported that soluble solids content (SSC) and titratable acidity (TA) of ‘Hayward’ kiwifruit are 12–15% and 0.8–1.0% respectively when the fruit soften to an eating-ripe firmness of 4.0–6.0 N.^7^ Compared with ‘Hayward’, ‘Hort16A’ kiwifruit has higher sweetness (SSC = 14–16%) and lower acidity (TA = 1.0–1.2%) when the firmness is suitable for eating at 4.5–6.0 N.^8^ Aroma of kiwifruit is the result of a subtle mixture volatile compounds, which is also a crucial factor contributed to consumer acceptance.^9^ Wang *et al.* identified the volatile compounds released from ‘Hayward’ and ‘Hort16A’ at different ripening stages by means of gas chromatography-mass spectrometry (GC-MS).^10^ They found that straight-chain aldehydes and esters are the dominant compounds in the two cultivars, but differences do exist. ‘Hayward’ kiwifruit has more green note compounds, *e.g.* hex-*E*2-enal and hexanal, while ‘Hort16A’ kiwifruit has more tropic note compounds, *e.g.* ethyl butanoate, eucalyptol, and methyl sulfanyl. However, no research has been found about the flavor quality of red-fleshed kiwifruit.

As we know, the traditional methods for fruit flavor detection mainly consist of 3 aspects: traditional chemical analysis, high-end instrumental analysis, and sensory evaluation by human panel. However, they are usually time-consuming and low-efficiency.^11^ The development of technology has promoted some rapid and non-destructive techniques that could be used to trace the flavor of fruit. Inspired by the way human recognize samples *via* olfaction, electronic nose (E-nose) has been proven to be a good approach to achieve this. E-nose includes an array of gas sensors that respond sensitively to simple or complex odors, which has been reported as a rapid and non-destructive alternative for quality detection in food industry based on the assessment of aroma.^12^ Recently, some publications have reported the applications of E-nose technique in fruit quality control, such as cultivar classification,^13^ freshness discrimination,^14^ shelf-life monitoring,^15^ and postharvest treatment discrimination,^16^ but few of them focused on the quality detection of kiwifruit. Du *et al.* used a MOS E-nose system to predict the ripeness of kiwifruit, and results showed overall ripeness, SSC, and firmness were well predicted by E-nose combined with chemometrics.^17^ The above studies have illustrated the potential of E-nose technique in detecting the quality of fruit, but few articles have been published in evaluating the flavor quality of fruit by E-nose technology. As mentioned above, the aroma of fruit is a result of complex volatile compounds, and it may have tight relationships with internal quality. However, E-nose only gives the global response of samples' overall flavor profile, little is known about the inherent correlations between volatile compounds, quality indices, and E-nose response.

In this study, internal quality and aroma of a red-fleshed ‘Hongyang’ kiwifruit during ripening were investigated by GC-MS and E-nose, and the potential of E-nose in tracing kiwifruit flavor quality was attempted. The main objectives of this study were: (1) to investigate quality indices and volatile compounds of ‘Hongyang’ kiwifruit during ripening; (2) to explore the potential correlations between quality indices, volatile compounds, and E-nose data based on ANOVA partial least squares regression (APLSR); (3) to predict the quality indices by E-nose combined with chemometrics.

## 2 Materials and methods

### 2.1 Kiwifruit samples

The red-fleshed kiwifruit (*Actinidia chinensis* Planch. var. rufopulpa [C. F. Liang and R. H. Huang] C. F. Liang and A. R. Ferguson) cultivar called ‘Hongyang’ was investigated in this study. Kiwifruit were hand harvested from a local orchard located in Shaoxing (120°51′ E, 29°49′ N), China, on September 2, 2018. One hundred samples were selected according to the approximately uniform size and weight. Because the fruit were harvested from different trees, then pooled and randomized, the experimental design was completely randomized. Once arrived at the laboratory in Zhejiang University, Hangzhou, the fruit were stored in an incubator (STIK (Shanghai) CO., China) for ripening at controlled natural conditions (20 °C and 70% relative humidity). The postharvest storage lasted for two weeks that covered the whole ripening stages from unripe, mid-ripe, eating-ripe, to over-ripe. Samples from the incubator were analyzed every 3 days, and there were 5 groups in the investigation: day0, day3, day6, day9, and day12 (day3 is the third day since harvest, and day0 is the harvested day).

### 2.2 Physicochemical determination

Firmness, sweetness, and acidity are three of the most important indices to the internal quality of kiwifruit related to consumer preference.^6^

Firmness was measured by M–T puncture test using a Universal Testing Machine (Instron 5543, Instron Corp., USA).^18^ A 6 mm cylindrical probe was used to puncture the samples at a speed of 20 mm min^−1^. The penetration depth was 8 mm, and three sites were tested along the equatorial plane with 120° intervals. The maximum force in penetration was acquired to evaluate the firmness of kiwifruit.^19^ The obtained values at three sites were averaged to represent the overall firmness for each sample.

Sweetness and acidity were measured by the juice of kiwifruit. After the M–T puncture test, the kiwifruit sample was squeezed into juice by a blender (DESIGNER 675, Blendtec, USA), and the juice was filtered through 4 layers of 120 mesh cotton in order to remove the solid particles. SSC, expressed as the percentage of sugars (%), was measured by a digital pocket refractometer (PAL-1, Atago Co., Ltd., Japan) to indicate the sweetness of kiwifruit. TA, expressed as the concentration of citric acid (%), was measured by titration to pH 8.1 with 0.1 mol L^−1^ NaOH to indicate the acidity of kiwifruit.^20^ The ratio of SSC to TA (SSC/TA ratio) that is a comprehensive attribute descriptor to the flavor of kiwifruit, was also calculated.^21^ For determination of firmness, sweetness, and acidity, triplicates were carried out for each group, and the results were expressed as mean values and stand deviations.

### 2.3 HS-SPME and GC-MS measurement

Head space-solid phase microextraction (HS-SPME) is a very effective analytical technique commonly used for concentrating fruit volatiles.^22^ In this study, volatiles from the kiwifruit were collected from combined juices at the same ripening time, following the method described by Wang *et al.*^10^ A commercial fiber coated with divinylbenzene/carboxen/polydimethylsiloxane (DVB/CAR/PDMS; Supelco, Inc., USA) was used to extract the headspace volatiles. For each sampling extraction, a vial (volume, 10 mL) with 5 mL kiwifruit juice and 1.0 g sodium chloride was sealed by a polypropylene cap with a polytetrafluoroethylene/silicon septum (Supelco, Inc., USA). Octyl acetate was taken as the internal standard, and 10 μL of octyl acetate in hexane (1 μL L^−1^) was injected into the juice by a syringe.^23^ The juice liquid was magnetically stirred at 40 °C (400 rpm) for 5 min before the headspace concentration. After homogenization, the fiber was exposed to the headspace for 60 min at 40 °C. Afterwards, the fiber was pulled into the needle sheath, removed from the vial, and inserted into the injection port of GC system for 8 min at 250 °C for thermal desorption. Finally, the volatile compounds of samples with triplicates were fingerprinted by means of GC-MS.

An Agilent 7890A Network for GC (Agilent Technologies, USA) coupled with an Agilent 5973 Network detector for MS (Agilent Technologies, USA) was employed to detect the volatile compounds. The GC-MS system was equipped with the NIST spectral library (NIST 11.0, National Institute of Standards and Technology, USA). Confirmation was accomplished by comparing the recorded mass spectra with the reference data from NIST spectral library. Semi-quantification of volatile compounds was performed by comparing the areas of the peaks to that of the internal standard of octyl acetate. The conditions and parameters of GC-MS were optimized by pre-experiments, and the results were as follows:

(1) GC conditions: an HP-5MS methyl siloxane chromatographic column (30 m × 250 μm × 0.25 μm; Agilent Technologies, USA) was used for GC separations. Helium was used as the carrier gas with a flow rate of 24 mL min^−1^. The temperature in the GC injector was kept at 250 °C. While the temperature in the GC oven was programmed as follows: it was initially kept at 35 °C for 3 min, then increased to 45 °C at the rate of 3 °C min^−1^, next heated to 120 °C at 2 °C min^−1^, and finally ramped up to 240 °C at 6 °C min^−1^.

(2) MS conditions: electron impact (EI) ionization source; electron energy of 70 eV; ionization temperature at 230 °C; quadrupole temperature at 150 °C; interface temperature at 280 °C; and quantity scanning ranged from 30 to 500 amu.

### 2.4 E-nose detection

A portable PEN3 E-nose system (Airsense Analytics GmBH, Germany) was used to characterize the kiwifruit. The E-nose has an array of 10 different metal oxide semiconductor (MOS) gas sensors in the chamber. Each of these sensors is sensitive to specific volatile compositions, and Table 1 describes the detailed characteristics of these 10 MOS gas sensors.

**Table 1:** Sensors in PEN3 E-nose system and their sensitivity description

Number	Name	Sensitive substances	Reference
S1	W1C	Aromatic compounds	Toluene, 10 ppm
S2	W5S	Very sensitive, broad range sensitivity, react on nitrogen oxides, very sensitive with negative signal	NO_2_, 1 ppm
S3	W3C	Ammonia, used as sensor for aromatic compounds	Propane, 1 ppm
S4	W6S	Mainly hydrogen, selectively (breath gases)	H_2_, 100 ppb
S5	W5C	Alkanes, aromatic compounds, less polar compounds	Propane, 1 ppm
S6	W1S	Sensitive to methane (environment) *ca.* 10 ppm. Broad range, similar to no. 8	CH_3_, 100 ppm
S7	W1W	Reacts on sulphur compounds, H_2_S 0.1 ppm. Otherwise sensitive to many terpenes and sulphur organic compounds, which are important for smell, limonene, pyrazine	H_2_S, 1 ppm
S8	W2S	Detects alcohol's, partially aromatic compounds, broad range	CO, 100 ppm
S9	W2W	Aromatics compounds, sulphur organic compounds	H_2_S, 1 ppm
S10	W3S	Reacts on high concentrations > 100 ppm, sometime very selective (methane)	CH_3_, 10CH_3_, 100 ppm

Before the E-nose measurement, each individual kiwifruit was put in a 500 mL glass beaker for one hour to accumulate the headspace concentration. The beaker was sealed with plastic wrap, and a piece of elastic band was covered under the wrap to ensure a good seal. In the measurement, the E-nose sampling needle was inserted into the wrap to collect kiwifruit headspace gas. Each measurement lasted for 2.5 min, including 90 s measurement process and 60 s cleaning process. The sample gas was pumped into the sensor chamber with a flow rate of 200 mL min^−1^, and the signals per second were collected. Cleaning gas, which was used as the reference gas, was pumped into the sample gas path to normalize the sensor signals. All the E-nose measurements were carried out at a controlled temperature of 20 °C ± 0.5 °C and a relative humidity of 70% ± 5%.

### 2.5 Data analysis

Principal component analysis (PCA) is a very powerful multivariate statistic method used to analyze the inherent structure of data. The main purpose of this linear feature extraction method is to reduce dimensions, projecting the *m*-dimensional data set in a smaller dimension. Linear discrimination analysis (LDA) is another widely used statistic method to find new lower-dimensional variables with good discrimination from the original data set. Compared with PCA, the LDA method is supervised, which can notice the distribution of points in the same category and the distance between them. The graphical view of LDA analysis is similar to PCA displays.^24^

Partial least squares regression (PLSR) is a multi-linear regression method to find a linear relationship between responses and independent variables. PLSR combines the advantages of principle component analysis (PCA), multiple linear regression (MLR), and analysis of variance (ANOVA), which is appropriate for solving multicollinearity issues. The modified jack-knifing was applied to estimate the uncertainty variance of the regression coefficient (*P* < 0.05) and to extract significant indications determined in the quantitative APLSR.^11^ Support vector machine (SVM) is a linear machine working in the high dimensional feature space formed by the non-linear mapping of the *n*-dimensional input vector into a *K*-dimensional feature space (*K* > *n*). The specialized learning procedures in SVM neural network are helpful in obtaining the global minimum of the error function and excellent generalization ability of the trained network. SVM performs well on problems with small samples of non-linear and high-dimensional data, just like the E-nose data.^25^

In this study, there were 5 groups in the E-nose measurement, each group contained 20 replicates, and in total 100 samples made up the E-nose data set. In data modeling, the data set was divided into two subsets: 15 samples of each group were randomly selected as the training set and 5 samples of each group were considered as the testing set. PCA and stepwise LDA were used for visualization of E-nose data. APLSR was applied to analyze the correlations between quality indices, volatile compounds, and E-nose data. PLSR and SVM were employed for the quantitative prediction of quality indices. Prediction performances were evaluated by two parameters from the fitted equations: square correlation coefficient (*R*^2^) and root mean square error (RMSE). The larger *R*^2^ and the lower RMSE would indicate the better prediction performance.^12^

The data processing methods of PCA and LDA were performed by Statistical Product and Service Solutions v22.0 (International Business Machines Corporation, USA). PLSR and APLSR were performed using the Unscrambler X (CAMO ASA, Trondheim, Norway). SVM algorithms were run in MATLAB 2014b software (MathWorks, USA).

## 3 Results and discussion

### 3.1 Results of quality indices and volatile compounds during ripening

#### 3.1.1 Quality indices

Physicochemical indices related to the internal quality of ‘Hongyang’ kiwifruit during ripening were detected for two weeks with 3 day intervals, and results of firmness, SSC, TA, and SSC/TA ratio are presented in Table 2. The quality indices obviously changed during ripening, and the changes could be mainly divided into three phases. For firmness, the values kept at a high level at the beginning of ripening (from day0 to day3). Then the firmness had a rapid decrease from day3 to day9, followed by a slow softening phase at the last of ripening (from day9 to day12). 3–6 N was considered as the eating-ripe firmness for kiwifruit, and the firmness less than 3 N would be considered over-ripe.^10^ The firmness of ‘Hongyang’ kiwifruit had a similar changing tendency with that of ‘Hayward’ and ‘Hort16A’ kiwifruit. But the softening time of ‘Hongyang’ kiwifruit was much shorter than the other two cultivars.^26^ As for the reason, the softening was influenced by the differences of cell wall composition, gene expression, and enzyme activity in different genotypes.^27^ SSC and TA had the opposite changes during ripening. An increase and a decrease occurred separately for SSC and TA very slowly at the first phase. The changes became sharp at the second phase and then went slowly at the last phase. SSC/TA ratio is an index representing the overall sweetness and acidity of kiwifruit, which could reflect the acceptance for consumers with regard to the taste. After a slow increase in the first 3 days and a rapid increase in the following 6 days, SSC/TA ratio became 22.63 at an eating-ripe condition (day9) and 23.76 at an over-ripe condition (day12). Over-ripe kiwifruit contain more sugars and a higher diversity of organic acids, which is preferable for wine production but not acceptable for eating.^28^ The changes of quality indices made kiwifruit become increasingly soft, sweet, and less tart during ripening. Similar results had been confirmed by Wang *et al.* that the mean soluble solids concentration of ‘Hongyang’ kiwifruit could be as high as 19.6%, along with a low total acid content of 0.49%.^29^ It was reported that ‘Hongyang’ kiwifruit had the highest sugar to acid ratio among all varieties of kiwifruit.^30^

**Table 2:** Results of physicochemical indices related to internal quality of ‘Hongyang’ kiwifruit during ripening^a^

Ripening time	Day0	Day3	Day6	Day9	Day12
Firmness (*N*)	48.35 ± 1.51^a^	53.84 ± 4.16^a^	27.48 ± 4.57^b^	3.25 ± 0.74^c^	2.86 ± 0.40^c^
SSC (%)	8.91 ± 2.01^b^	9.26 ± 2.07^b^	17.62 ± 1.16^a^	20.54 ± 1.25^a^	19.94 ± 0.54^a^
TA (%)	1.10 ± 0.10^a^	1.09 ± 0.13^a,b^	0.95 ± 0.09^a,b^	0.91 ± 0.01^a,b^	0.85 ± 0.08^b^
SSC/TA ratio	8.10 ± 1.76^b^	8.71 ± 2.84^b^	18.61 ± 2.41^a^	22.63 ± 1.29^a^	23.76 ± 2.69^a^

a Results are expressed as mean values ± stand deviations (*n* = 3 for each group). Means in the same row followed by different inline letters (a, b, and c) are statistically different by the Tukey's HSD test (*P* < 0.05).

#### 3.1.2 Volatile compounds

Volatile compounds of ‘Hongyang’ kiwifruit during ripening were analyzed by GC-MS after the physicochemical detection, and results of the main constituents are presented in Table 3. More than 20 volatile compounds were identified in ‘Hongyang’ kiwifruit, whose dominant compounds were straight-chain aldehydes and esters. Most volatile compounds had been identified in the previous researches on the two commercial cultivars *A. deliciosa* ‘Hayward’ and *A. chinensis* ‘Hort16A’, but certain specific volatile compounds had some differences.^5^

**Table 3:** Volatile compounds (μg kg^−1^) of ‘Hongyang’ kiwifruit during ripening^a^^,^^b^

No.	Constituents	Ripening time				
		Day0	Day3	Day6	Day9	Day12
1	(*E*)-2-Hexenal	119.00 ± 17.47^a^	49.29 ± 3.11^b^	20.78 ± 4.41^c^	20.77 ± 4.26^c^	21.62 ± 8.85^c^
2	(*E*,*E*)-2,4-Hexadienal	0.21 ± 0.01^b^	0.35 ± 0.13^a,b^	0.32 ± 0.10^a,b^	0.14 ± 0.05^b^	0.56 ± 0.11^a^
3	Octanal	ND	0.02 ± 0.01^b^	ND	0.24 ± 0.12^a^	ND
4	Hexanal	10.92 ± 0.10^a^	10.74 ± 0.42^a^	9.26 ± 0.67^a^	10.00 ± 0.79^a^	3.49 ± 1.01^b^
5	Nonanal	ND	1.60 ± 0.39^a^	ND	1.45 ± 0.36^a^	1.47 ± 0.62^a^
6	Decanal	1.05 ± 0.95^b^	ND	ND	2.94 ± 0.65^a^	1.82 ± 0.38^a,b^
7	Benzaldehyde	ND	ND	0.66 ± 0.08^a,b^	1.10 ± 0.16^a^	1.00 ± 0.22^a^
8	d-Limonene	ND	0.74 ± 0.10^a^	0.79 ± 0.09^a^	0.54 ± 0.14^a,b^	0.66 ± 0.04^a^
9	Terpinolene	ND	0.13 ± 0.04^a^	0.12 ± 0.05^a^	0.09 ± 0.03^a^	0.10 ± 0.02^a^
10	γ-Terpinene	ND	0.10 ± 0.03^a^	ND	0.08 ± 0.02^a^	0.07 ± 0.01^a^
11	Eucalyptol	1.46 ± 0.52^b^	6.05 ± 0.54^a^	6.79 ± 0.27^a^	6.91 ± 0.50^a^	6.08 ± 0.28^a^
12	(*E*)-2-Hexen-1-ol	ND	1.03 ± 0.21	ND	ND	ND
13	(*Z*)-2-Hexen-1-ol	ND	1.16 ± 0.16	ND	ND	ND
14	Methyl butanoate	ND	ND	1.49 ± 0.22^b^	12.72 ± 1.21^a^	14.26 ± 0.55^a^
15	Methyl benzoate	ND	ND	0.33 ± 0.05^c^	6.88 ± 0.40^a^	3.92 ± 0.31^b^
16	Methyl hexanoate	ND	ND	1.26 ± 0.27^c^	3.27 ± 0.23^a^	2.26 ± 0.08^b^
17	Ethyl butyrate	ND	ND	ND	10.04 ± 0.65^a^	6.87 ± 0.16^b^
18	Ethyl hexanoate	ND	ND	ND	9.51 ± 0.58^a^	7.45 ± 0.45^b^
19	Methyl salicylate	ND	ND	ND	0.52 ± 0.05	ND
20	Ethyl benzoate	ND	ND	ND	0.42 ± 0.07	ND
21	Isobutyl hexanoate	ND	ND	ND	0.29 ± 0.03	ND

a ND, not identified.

b Results are expressed as mean values ± stand deviations (*n* = 3 for each group). Means in the same row, followed by the same letter, or without a letter, are not significantly different (*P* < 0.05), determined by the Tukey's HSD test.

Aldehydes are important contributors to the aroma volatiles of kiwifruit, being responsible for the fresh, grassy, and green notes perceived by consumers.^31^ Apart from the two most abundant aldehyde compounds of (*E*)-2-hexenal and hexanal, (*E*,*E*)-2,4-hexadienal was detected in ‘Hongyang’ kiwifruit which has not been reported in other kiwifruit cultivars. On the other hand, esters are the compounds mainly responsible for the sweet and fruity notes of kiwifruit.^32^ Methyl butanoate, methyl benzoate, methyl hexanoate, and ethyl butyrate were detected as the major ester compounds in ‘Hongyang’ kiwifruit, and more esters were identified in the ripe kiwifruit. In addition, the mint-like eucalyptol, as a representative of alcohols, was found at a relatively high level. Besides these compounds, some terpenes like d-limonene, terpinolene, and γ-terpinene were also identified in ‘Hongyang’ kiwifruit. It could be seen that ‘Hongyang’ kiwifruit shared a number of volatile compounds in common with ‘Hayward’ and ‘Hort16A’ kiwifruit. But some volatile compounds like (*E*,*E*)-2,4-hexadienal, terpinolene, γ-terpinene, methyl salicylate, and isobutyl hexanoate were uniquely identified, which might be the volatile biomarkers for ‘Hongyang’ kiwifruit.

Taking the ripening time into consideration, the volatile compounds detected in kiwifruit were dynamically changing during ripening. At the beginning, the fruit aroma volatiles were mainly composed of aldehydes, especially referring to (*E*)-2-hexenal and hexanal. With the increment of ripening time, the aldehydes declined. In particular, (*E*)-2-hexenal decreased to a relatively low level of about 20 μg kg^−1^ at day6, then kept at this level in the following period. On the other hand, the fruity esters had an increase during ripening. For example, no methyl butanoate was detected at the beginning of ripening. Then methyl butanoate was initially detected at day6 with 1.49 μg kg^−1^, and the content increased to 12.72 μg kg^−1^ at day9 and to 14.26 μg kg^−1^ at day12. In addition, eucalyptol had an overall increase during the whole ripening period. However, there was no obvious difference for the terpenes among different ripening days. The previous publications confirmed these changes that an increase in esters and alcohols and a decrease in the proportion of aldehydes were observed during kiwifruit ripening.^10,33^ The changes of volatile compounds were explained by the gene regulation that the reduction of aldehydes was the result of diminished lipoxygenase (LOX) activity and the biosynthesis of esters was strongly upregulated by the action of alcohol acyl transferases (AATs) during ripening.^34^

#### 3.1.3 Correlations of quality indices with volatile compounds

**Fig. 1:**
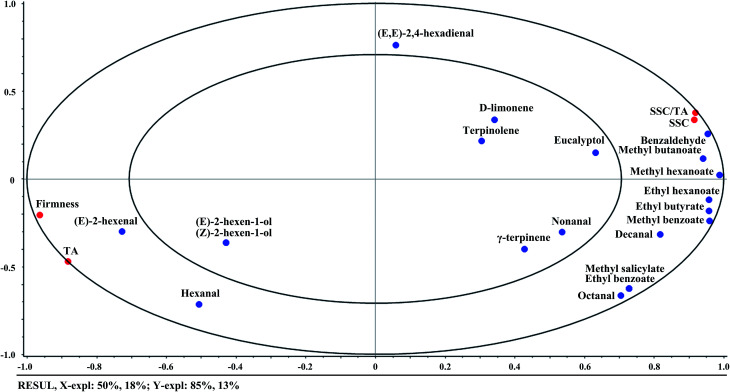
Correlation loadings plot for PC1 *versus* PC2. The model was derived from volatile compounds as the *X*-matrix (blue circles) and quality indices as the *Y*-matrix (red circles). The small and big ellipses represent *R*^2^ = 50 and 100%, respectively.

APLSR was applied to study the correlations between quality indices and volatile compounds. The *X*-matrix and *Y*-matrix were designated as the above volatile compounds and quality indices, respectively, and mean data across the measurement were used. The derived APLSR model included two significant PCs explaining 68% of the variance in *X*-matrix (volatile compounds) and 98% of that in *Y*-matrix (quality indices). The small ellipse indicated 50% of the explained variance and the big one explained 100% of the variance. The variables inside the inner ellipse could be considered poorly explained by the first two components, and the variables between the small and big ellipses were well explained. The resultant correlation loadings plot of the first two components (PC1 and PC2) is shown in Fig. 1. It indicated that (*E*)-2-hexen-1-ol, (*Z*)-2-hexen-1-ol, d-limonene, terpinolene, γ-terpinene, nonanal, and eucalyptol were located outside the small ellipse, which were poorly explained. SSC, SSC/TA, and all ester compounds were located on the right side in PC1, while firmness, TA, and the main aldehyde compounds were located on the left side. The shorter the distance between two variables, the higher the correlation of variables with each other. It could be noticed that firmness and TA had highly positive correlations with (*E*)-2-hexenal and hexanal that were abundant in unripe fruit. On the other hand, SSC and SSC/TA ratio had highly positive correlations with ester compounds that were accumulated in ripe fruit, especially referring to methyl butanoate, methyl hexanoate, ethyl hexanoate, ethyl butyrate, and methyl benzoate. The positive correlations between firmness and aldehyde compounds, SSC and ester compounds were also found in peach fruit.^35^ It demonstrated that the aroma had substantial correlations with internal quality of fruit during ripening.

### 3.2 Results of E-nose

#### 3.2.1 Responses of E-nose

**Fig. 2:**
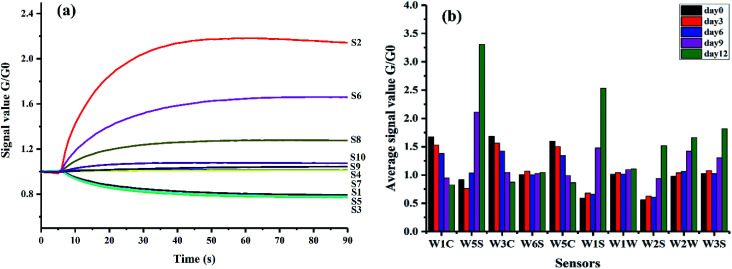
Responses of E-nose to the aroma of kiwifruit: (a) a typical E-nose response of 10 sensor curves during 90 s measurement and (b) mean values of sensor responses at the 80th second obtained from samples at different ripening times.

Fig. 2(a) showed a typical E-nose response made of 10 sensor curves during 90 s measurement to the aroma of kiwifruit. In Fig. 2(a), the *X*-axis represented the time and the *Y*-axis represented the sensor signals. The signals were expressed by *G*/*G*0, where *G* and *G*0 represented the conductivities of sensors in the sample gas and in the reference gas, respectively. Each curve represented the change of a sensor's ratio of conductivities during the measurement. It could be seen that the signal of each sensor was low in the initial period, then changed rapidly during the 5th–60th second, and finally came to a dynamic balance after the 60th second. In this study, the response values of sensors at the 80th second which were located at the stable phase were extracted as the features in the following analysis.

Mean values of sensor responses at the 80th second were calculated to explore the differences of E-nose signals among different sample groups relating to the ripening time in Fig. 2(b). Differences of the mean values could be observed among different groups. Samples at day9 and day12 had clear differences compared with the other groups. It could be attributed to the obvious changes of volatile compounds of kiwifruit after day9 as illustrated above. All the gas sensors presented the sensitivity to the changes of ripening time, especially sensors S2 and S6. It may be because these two sensors were very sensitive to aromatic compounds and with broad range sensitivity as described in Table 1. Slight changes of the aroma during ripening further accumulated the obvious changes of sensor signals. The results of mean values indicated the potential to distinguish samples at different ripening times by E-nose data.

#### 3.2.2 Results of PCA and LDA

**Fig. 3:**
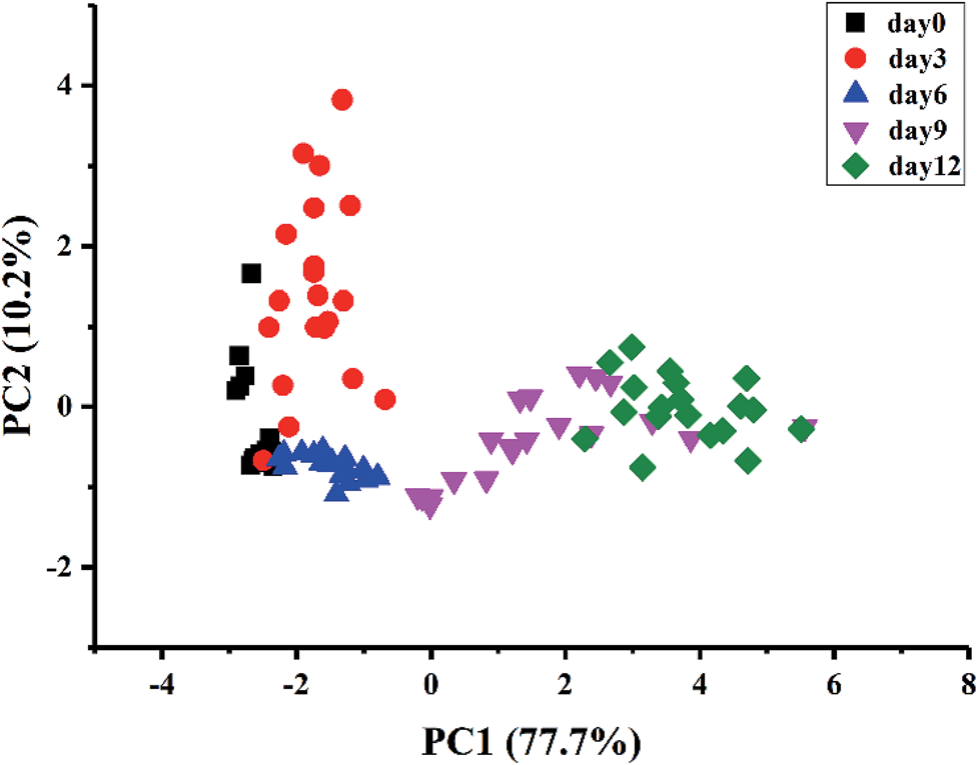
Visualization of the distribution of kiwifruit samples by PCA.

The distribution of kiwifruit samples at different ripening times was visualized by PCA (Fig. 3). The PCA results showed that the first two principal components explained 87.9% of the total variance (PC1 = 77.7% and PC2 = 10.2%). Although samples at day9 and day12 could be discriminated from the other groups, the samples at day0, day3, and day6 were overlapped and the samples at day9 and day12 were overlapped. The results could be explained by the clear differences of E-nose responses to the samples at day9 and day12 compared with the other groups as described above. Intrinsically, the internal quality and aroma of kiwifruit had noticeable changes during ripening, especially when the fruit began to be ripe.

**Fig. 4:**
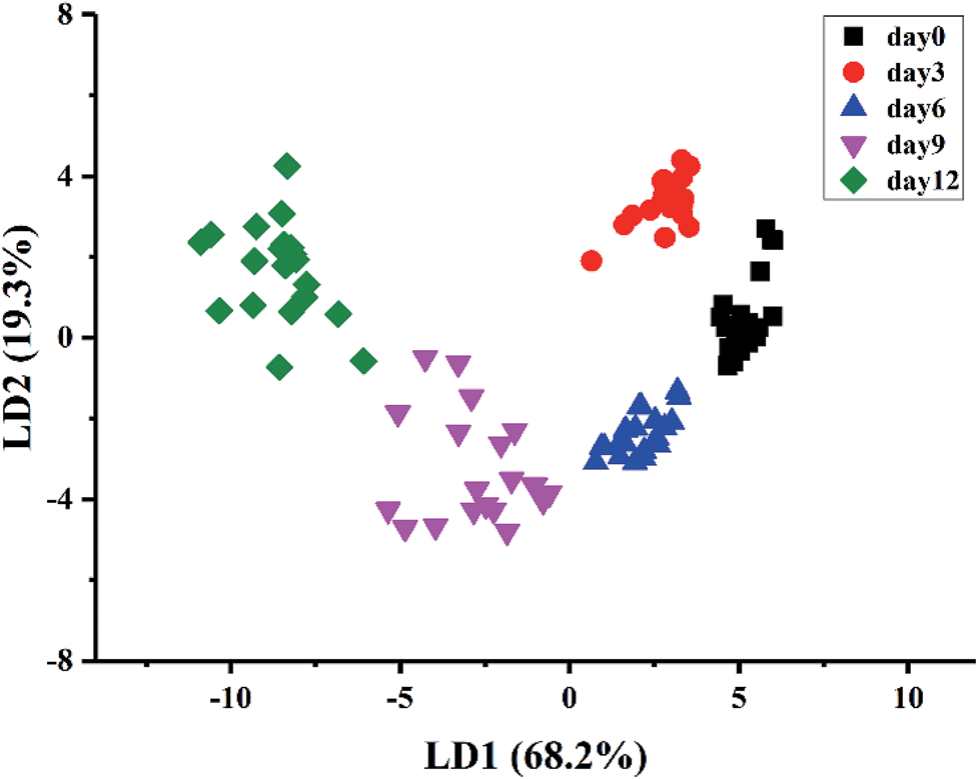
Discrimination of kiwifruit samples by LDA.

A stepwise LDA procedure with leave-one-out cross validation was applied to discriminate the kiwifruit at different ripening times. During the stepwise modeling, variables were included if *F* < 0.05 and variables were removed if *F* > 0.10. Wilks' Lambda test was carried out to confirm which discriminant function was significant. The LDA results (Fig. 4) showed that the sum of LD1 and LD2 explained 87.5% of the total variance (LD1 = 68.2% and LD2 = 19.3%). It could be seen that the samples could be well classified into five groups according to their ripening times. The discrimination results of the samples at day0, day3, and day6, as well as the samples at day9 and day12, were improved compared with PCA. As a result, 100% of the samples are correctly classified for the original procedure and 98% for the cross-validation step. The above results demonstrated that the kiwifruit at different ripening times could be discriminated by E-nose data, indicating the potential application of E-nose technique to trace the ripeness of fruit.

#### 3.2.3 Correlations of E-nose data with volatile compounds

**Fig. 5:**
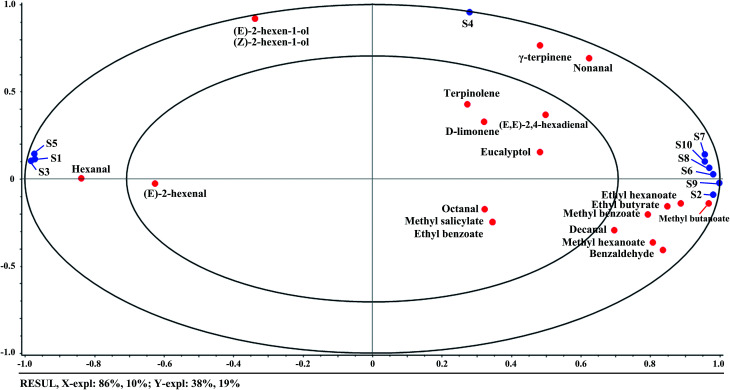
Correlation loadings plot for E-nose data (*X*-matrix) and volatile compounds (*Y*-matrix). The concentric small and big ellipses showed the locus of 50 and 100% explained variance.

Correlations between E-nose data and volatile compounds were also analyzed by APLSR. Fig. 5 showed a first and second factor loadings plot for E-nose data (*X*-matrix) and GC-MS volatile compounds (*Y*-matrix), where the E-nose sensors were mainly clustered into two groups except for S4. The first sensor cluster (S7, S10, S8, S6, S9, and S2) was located on the right side of the coordinate system, while the second sensor cluster (S1, S3, and S5) was located on the left side. Closing to the first sensor cluster, there were ethyl hexanoate, ethyl butyrate, methyl benzoate, methyl butanoate, methyl hexanoate, and benzaldehyde, indicating the responses of the first sensor cluster had positive correlations with these compounds. The close distance between the second sensor cluster and hexanal indicated the sensors (S1, S3, and S5) were positively correlated with the green note compounds of kiwifruit. On the other hand, it seemed that S4 was mainly correlated with terpene compounds such as γ-terpinene, terpinolene, and d-limonene according to the close distances. However, some volatile compounds, such as (*E*,*E*)-2,4-hexadienal, eucalyptol, octanal, methyl salicylate, and ethyl benzoate, were placed in between the above two sensor clusters, which seemed having no direct relationships with E-nose data. As for reasons, the variance explained by the first principal component accounted for 86% of E-nose data, but the volatile compounds related variances were only extracted for 10%, which may lead to losing some information of volatile compounds. Other guesses might be that these compounds had non-linear relationships with the E-nose data or the sensors had cross sensitivity to volatile compounds. The results demonstrated that the E-nose data were correspondingly correlated to volatile compounds, which may further be used to trace the quality indices of kiwifruit during ripening.

### 3.3 Prediction of quality indices by E-nose data

#### 3.3.1 Prediction results based on PLSR

**Fig. 6:**
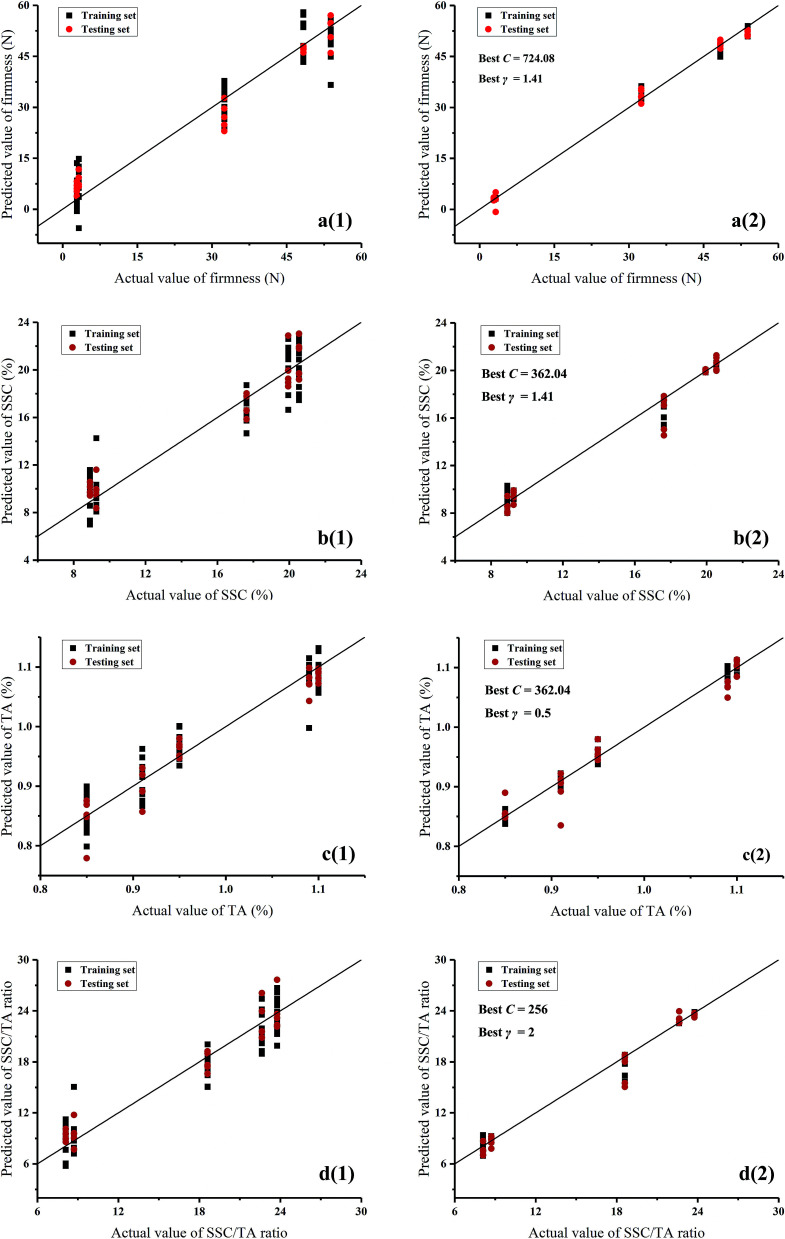
Predicted *versus* actual values from PLSR and SVM models: (1) presents PLSR and (2) presents SVM; (a) stands for firmness, (b) stands for SSC, (c) stands for TA, and (d) stands for SSC/TA ratio.

PLSR is a multivariate linear regression method which was used to establish the correlations between E-nose data and quality indices (firmness, SSC, TA, and SSC/TA ratio). Fig. 6(a–d)(1) visualized the linear relationships between the predicted and actual values of quality indices based on PLSR. It could be seen that the firmness, SSC, TA, and SSC/TA ratio were overall predicted well by E-nose data. The evaluating parameters of *R*^2^ and RMSE in training and testing sets are listed in Table 4. The results showed a good correlation between E-nose data and quality indices (*R*^2^ > 0.90 in the training and testing sets). However, E-nose data presented less inferior correlations with TA than that with SSC, firmness, and SSC/TA ratio. It may be explained from Fig. 1 that SSC and SSC/TA ratio had positive correlations with each other, and negatively correlated with firmness. But the far distance between firmness and TA indicated the inferior correlations of TA with the other three indices. The prediction results based on PLSR quite agreed with the results of correlation analysis above.

**Table 4:** Results of evaluating parameters for prediction models based on PLSR and SVM

Algorithms	Quality indices	Training set		Testing set	
		*R* ^2^	RMSE	*R* ^2^	RMSE
PLSR	Firmness	0.9207	6.1007	0.9439	5.1904
	SSC	0.9228	1.6118	0.9405	1.3071
	TA	0.9013	0.0276	0.9387	0.0252
	SSC/TA ratio	0.9158	1.9503	0.9448	1.6288
SVM	Firmness	0.9980	0.9662	0.9945	1.6347
	SSC	0.9907	0.5025	0.9668	0.9201
	TA	0.9885	0.0107	0.9454	0.0223
	SSC/TA ratio	0.9914	0.6312	0.9775	1.0941

#### 3.3.2 Prediction results based on SVM

SVM is a supervised non-linear learning regression method which was also employed to establish the correlations between E-nose data and quality indices. Radial basis function (RBF) was chosen as the core function and 5-fold cross-validation was applied in SVM. A grid search method was used to optimize the penalty parameter *C* and kernel parameter *γ* with exponentially growing sequences. Each combination of *C* and *γ* was checked by 5-fold cross-validation until the best cross-validation MSE (CV_mse_) was obtained. Fig. 6(a–d)(2) visualized the linear relationships between the predicted and actual values of quality indices based on SVM. The prediction results of firmness, SSC, TA, and SSC/TA ratio based on SVM were greatly improved compared with those based on PLSR. The evaluating parameters of *R*^2^ and RMSE in training and testing sets are listed in Table 4. The results verified the improvements as observed in plots that the regression models based on SVM showed a good performance in predicting quality indices (*R*^2^ > 0.99 in the training set and *R*^2^ > 0.96 in the testing set except for TA). It illustrated that the SVM algorithm was able to extract useful information more effectively than PLSR in processing the E-nose data that correlated to quality indices. In addition, the correlations between E-nose data and quality indices may be more complicated with non-linearity.

## 4 Conclusions

This study attempted to trace the internal quality and aroma of a red-fleshed ‘Hongyang’ kiwifruit during ripening by GC-MS and E-nose. The results showed that (*E*,*E*)-2,4-hexadienal, terpinolene, γ-terpinene, methyl salicylate, and isobutyl hexanoate were identified as the unique volatile compounds in ‘Hongyang’ kiwifruit. With the increment of ripening time, the green note aldehydes declined, the main fruity esters increased, and the terpenes had no obvious changes. Firmness and TA had highly positive correlations with (*E*)-2-hexenal and hexanal, while SSC and SSC/TA ratio had positive correlations with methyl butanoate, methyl hexanoate, ethyl hexanoate, ethyl butyrate, and methyl benzoate. On the other hand, the E-nose sensors of S7, S10, S8, S6, S9, and S2 were positively correlated with ethyl hexanoate, ethyl butyrate, methyl benzoate, methyl butanoate, methyl hexanoate, and benzaldehyde, S1, S3, and S5 were mainly correlated with hexanal, and S4 was correlated with terpene compounds. The PCA and LDA results indicated that the samples at different ripening times could be discriminated by E-nose data. The prediction results based on PLSR and SVM revealed high correlations between E-nose data and quality indices. A better performance was achieved by SVM for the prediction of firmness, SSC, TA, and SSC/TA ratio (*R*^2^ > 0.98 in the training set and *R*^2^ > 0.94 in the testing set). The results of this study showed that the ‘Hongyang’ kiwifruit presented some specific characteristics of flavor quality. Furthermore, the E-nose technique could be used to trace the flavor quality of kiwifruit during ripening.

## Conflicts of interest

The authors declare that they have no conflicts of interest.

## Supplementary Material

## References

[cit1] Belrose Inc. , World kiwifruit review, 2011, Pullman, WA, USA, Belrose Inc

[cit2] Wang M., Li M., Meng A. (2003). Acta Hortic..

[cit3] Montefiori M., McGhie T. K., Costa G., Ferguson A. R. (2005). J. Agric. Food Chem..

[cit4] Kader A. A. (2008). J. Sci. Food Agric..

[cit5] Garcia C. V., Quek S.-Y., Stevenson R. J., Winz R. A. (2012). Trends Food Sci. Technol..

[cit6] Marsh K., Attanayake S., Walker S., Gunson A., Boldingh H., MacRae E. (2004). Postharvest Biol. Technol..

[cit7] Burdon J., Lallu N., Pidakala P., Barnett A. (2013). Postharvest Biol. Technol..

[cit8] Burdon J., Pidakala P., Martin P., McAtee P. A., Boldingh H. L., Hall A., Schaffer R. J. (2014). Postharvest Biol. Technol..

[cit9] Marsh K. B., Friel E. N., Gunson A., Lund C., MacRae E. (2006). Food Qual. Prefer..

[cit10] Wang M. Y., MacRae E., Wohlers M., Marsh K. (2011). Postharvest Biol. Technol..

[cit11] Qiu S., Wang J. (2015). J. Food Sci..

[cit12] Qiu S., Gao L., Wang J. (2015). J. Food Eng..

[cit13] Baietto M., Wilson A. D. (2015). Sensors.

[cit14] Hong X., Wang J. (2014). Anal. Methods.

[cit15] Xu S., Lü E., Lu H., Zhou Z., Wang Y., Yang J., Wang Y. (2016). Sensors.

[cit16] Qiu S., Wang J., Du D. (2017). Innovative Food Sci. Emerging Technol..

[cit17] Du D., Wang J., Wang B., Zhu L., Hong X. (2019). Sensors.

[cit18] Magness J. R. and Taylor G. F., USDA Agric. Cir., 1925, vol. 350

[cit19] Li H., Pidakala P., Billing D., Burdon J. (2016). Postharvest Biol. Technol..

[cit20] Benítez S., Achaerandio I., Sepulcre F., Pujolà M. (2013). Postharvest Biol. Technol..

[cit21] Qiu S., Wang J., Gao L. (2015). LWT--Food Sci. Technol..

[cit22] Yi J., Kebede B. T., Grauwet T., Van Loey A., Hu X., Hendrickx M. (2016). Postharvest Biol. Technol..

[cit23] Paterson V. J., Macrae E. A., Young H. (1991). J. Sci. Food Agric..

[cit24] Xu M., Ye L., Wang J., Wei Z., Cheng S. (2017). Postharvest Biol. Technol..

[cit25] Qiu S., Wang J., Tang C., Du D. (2015). J. Food Eng..

[cit26] Cheng C., Day S. (2013). Acta Hortic..

[cit27] Fullerton C. , PhD, The University of Auckland, 2011

[cit28] Towantakavanit K., Park Y., Gorinstein S. (2011). Cent. Eur. J. Biol..

[cit29] Wang M., Li M., Meng A. (2003). Acta Hortic..

[cit30] Li H., Zhu Y., Luo F., He H., Yuan H., Gao J., Zeng X., Huang C. (2015). J. Food Process. Preserv..

[cit31] Hatanaka A. (1993). Phytochemistry.

[cit32] Günther C. S., Matich A. J., Marsh K. B., Nicolau L. (2010). Phytochemistry.

[cit33] Bartley J. P., Schwede A. M. (1989). J. Agric. Food Chem..

[cit34] Nieuwenhuizen N. J. , Allan A. C. and Atkinson R. G., in The Kiwifruit Genome, ed. R. Testolin, H.-W. Huang and A. R. Ferguson, Springer International Publishing, Cham, 2016, pp. 135–147, 10.1007/978-3-319-32274-2_11

[cit35] Sánchez G., Besada C., Badenes M. L., Monforte A. J., Granell A. (2012). PLoS One.

